# Who Is Really at Risk for Anaphylaxis Due to COVID-19 Vaccine?

**DOI:** 10.3390/vaccines9010038

**Published:** 2021-01-11

**Authors:** Marco Caminati, Gabriella Guarnieri, Gianenrico Senna

**Affiliations:** 1 Department of Medicine, University of Verona and Verona University Hospital, Piazzale L.A. Scuro, 10, 37134 Verona, Italy; gianenrico.senna@aovr.veneto.it; 2 Respiratory Pathophysiology Unit, Department of Cardiac-Thoracic-Vascular Sciences and Public Health, University of Padova, 35121 Padova, Italy; gabriella.guarnieri@unipd.it; 3 Allergy Unit and Asthma Center, University of Verona and Verona University Hospital, 37134 Verona, Italy

The vaccination campaign against the Severe acute respiratory syndrome coronavirus 2 (Sars-Cov-2) started on 8 December 2020 in UK, after the approval of BNT162b2 by the Healthcare products Regulatory Agency (MHRA) [1]. The vaccine, produced by Pfizer and BioNTech, is a messenger ribonucleic acid (mRNA) [2]. Two severe cases of anaphylaxis were recorded on the second day of the vaccination program and involved two healthcare workers [3]. In the following days, an additional potential allergic reaction was described in the UK and eight further cases were reported in the US, always with the same vaccine [4]. The information raised some concern within the medical (and nonmedical) community and triggered a focused discussion on the causative mechanisms and the potential risk factors, in order to provide safety recommendations. Of note, the preliminary outputs from the scientific discussion are quite different in the UK and the US. In fact, the UK MHRA provisional regulation states, “any person with a history of anaphylaxis to a vaccine, medicine or food should not receive the Pfizer/BioNTech vaccine” [1]. On the contrary, according to the American Academy of Allergy and Clinical Immunology (AAAAI), one of the major scientific organizations in the field, “individuals with a history of food, pet, venom, environmental and latex allergy are able to proceed with vaccination with a standard 15-min observation. Those with a history of anaphylaxis to an injectable medication should use caution when receiving the vaccine and follow a 30-min observation period” [5]. Another renowned scientific academy, the American College of Allergy Asthma and Immunology (ACAAI) suggests: “Individuals with commune allergies (foods, inhalants, latex, insects) are no more likely than the general public to have an allergic reaction to the Pfizer-BioNTech vaccine. The Pfizer BioNTech Coronavirus Disease 2019 (COVID-19) vaccine should be administered in a health care setting where anaphylaxis can be treated. All the individuals must be observed for 20–30 min after the injection to monitor for any adverse reaction. All anaphylactic reactions should be managed immediately, with epinephrine as the first line treatment. Those with a history of severe anaphylaxis to any component of the Pfizer- BioNTech COVID-19 vaccination should not be vaccinated. The Pfizer BioNTech COVID-19 vaccine should not be administered to individuals with a history of polyethylene glycol as this is a component of this vaccine that is known to cause anaphylaxis” [6].

The application of MHRA recommendations would exclude from the vaccination campaign a not negligible proportion of subjects, who cannot be considered truly at risk according to the current evidence. Of note, the prevalence of anaphylaxis to any allergen is 3–5% in Western Countries [7], and the prevalence of self-reported food allergy, often not confirmed by a proper diagnostic work-up, is much higher [8]. Furthermore, hymenoptera venom hypersensitivity is not mentioned as a contraindication; nevertheless, its relevance in terms of prevalence in the adult population, which is at higher risk of Sars-Cov-2 infection than children or adolescents, is much more consistent when compared to food allergy [9]. In addition, hymenoptera venom allergy is frequently associated to mastocytosis, a condition characterized by a high risk of anaphylaxis [9]. That condition is not mentioned in any of the available documents. Both AAAAI and ACAAI provide similar recommendations [5,6]. However, ACAAI identifies previous anaphylaxis episodes due to injectable drugs, independently of their composition, as a specific risk factor. In addition, some additional differences are related to the waiting period after the vaccine administration.

When looking at previous statements on the topic, although not specific for COVID-19 vaccine, some issues deserve to be considered in the current frame as well. The Italian Healthcare Council identified severe asthma as a risk factor for vaccination-related adverse events and it recommended vaccines administration in a hospital setting for severe asthmatic patients [10]. A more restrictive position was expressed by the European Academy of Allergy Clinical Immunology (EAACI), which identified at risk patients only among individuals experiencing a previous allergic reaction to the same vaccine or to his preservatives [11]. Such a recommendation may not be completely applicable to the current situation, as we are facing a new disease and are using new vaccines. Nonetheless, up to now, anaphylaxis has been the only life-threatening condition reported during the vaccination campaign and therefore has to be properly managed and prevented. The identification of the culprit allergen as well as the mechanisms involved are still under investigation, though a potential involvement of polyethylene glycol has been suggested [12]. In the meantime, it is crucial that the vaccination campaign goes on, supported by an “empirical” yet reasonable safety protocol, relying on the known evidence on the topic. At risk patients might be identified in three categories:

1. Patients with previous documented hypersensitivity reactions to vaccines. BNT16b2 is different from the known vaccines but as a precautionary approach, administration in a hospital setting is advisable.
2. Patients affected by mastocytosis, with previous repeated episodes of anaphylaxis. Though previous studies did not identify it as a risk condition for vaccination, those patients deserve to receive vaccination in a hospital setting under specialist supervision, as previously stated [13].
3. Patients with severe asthma. Though no severe reaction to vaccines have been reported, a strong relationship has been observed between asthma and anaphylaxis, especially when asthma is not well-controlled [14]. For those patients, hospital administration is recommended.

At the moment a documented hypersensitivity to polyethylene glycol cannot be considered a specific contraindication to COVID-19 vaccine; its causative role in the reported anaphylaxis episode needs to be confirmed [12].

When hospital administration is recommended, some requirements need to be considered in terms of protocol and equipment. Besides epinephrine, a venous access and the availability of a resuscitation team are essential. The observation time may be at least 30 min, but the patients should have the possibility of a fast track to the hospital in case of necessity. The use of premedication protocols should be evaluated in specific cases.

Further research is needed in order to deeply investigate COVID-19 vaccines safety profile and to identify patient related risk factors, including specific pathological conditions affecting the immune system and potentially impacting on the vaccine outcomes [15,16,17]. *Vaccines* is running a Special Issue titled “When Vaccinations are Challenging: From Immune Diseases to Hypersensitivity Reactions”. Reviews and research contributions on COVID-19 vaccinations, as well as on the other topics listed in the Journal dedicated web page [18] are welcome.

However, despite the strong rationale supporting many anti-COVID-19 therapies, such as antivirals, cell-entry inhibitors, and immune-modulators, none of them have shown to be completely effective in all the affected individuals [17]. Under that perspective, preventive strategies are even more crucial, and more than one potential vaccine target against Sars-CoV-2 is currently under investigation, as a basis to develop universal vaccines against various new strains [19].

Anyway, in the meantime, the fear of hypersensitivity or adverse reactions should not overcome the worldwide need for carrying on the vaccination program, especially when considering that up to now the systemic allergic reactions were rare and the risk very low.
